# A comparative study of the clinical benefits of rivaroxaban and dabigatran in patients with nonvalvular atrial fibrillation with high bleeding risk

**DOI:** 10.3389/fcvm.2024.1445970

**Published:** 2024-09-17

**Authors:** Penghui Liu

**Affiliations:** Department of Cardiology, The Huai He Hospital of Henan University, Kaifeng, China

**Keywords:** rivaroxaban, dabigatran, nonvalvular atrial fibrillation, high bleeding risk, clinical benefits

## Abstract

**Objective:**

Rivaroxaban and dabigatran are approved to reduce the risk of stroke in patients with nonvalvular atrial fibrillation (NVAF). However, the clinical benefits of rivaroxaban and dabigatran in people with high bleeding risk are unclear.

**Methods:**

A retrospective study was conducted on NVAF patients admitted to the First Affiliated Hospital of Zhengzhou University from May 31, 2016 to May 31, 2019. These patients had a high risk of bleeding and were taking at least one study medication. The aim of the study was to evaluate clinical benefits by comparing the efficacy and safety risks of these two medications

**Results:**

A total of 1,301 patients with high bleeding risk were enrolled, including 787 patients in the rivaroxaban group and 514 patients in the dabigatran group. Results of the primary efficacy benefit endpoint were obtained from 104 patients (13.21%) in the rivaroxaban group and 81 (15.76%) patients in the dabigatran group [hazard ratio (HR): 0.860; 95% confidence interval (CI): 0.637–1.162; *P* = 0.327], this indicates that there was no significant difference between dabigatran and rivaroxaban in preventing stroke and systemic embolism in patients with high bleeding risk NVAF. The principal safety end points were observed in 49 (6.23%) patients in the rivaroxaban group and in 36 (7.00%) patients in the dabigatran group (HR: 0.801 in the rivaroxaban group; 95% CI: 0.512–1.255; *P* = 0.333), this indicates that there was no a significant difference in reducing fatal bleeding and critical organ bleeding. With respect to secondary efficacy and benefit endpoints, 28 (3.56%) patients in the rivaroxaban group and 26 (5.06%) patients in the dabigatran group died, with an HR of 0.725 (95% CI: 0.425–1.238; *P* = 0.239); 32 (4.07%) patients in the rivaroxaban group; and 31 (6.03%) patients in the dabigatran group had myocardial infarction (MI), with an HR of 0.668 (95% CI: 0.405–1.102, *P* = 0.114) in the rivaroxaban group, this indicates that there was no significant difference between dabigatran and rivaroxaban in preventing all-cause death and MI.

**Conclusions:**

In NVAF patients with high bleeding risk, there was no significant difference between dabigatran and rivaroxaban in preventing stroke and systemic embolism. There was also no significant difference between dabigatran and rivaroxaban in reducing fatal and critical organ bleeding.

**Clinical Trial Registration:**

Chinese Clinical Trials Registry, identifier ChiCTR2100052454.

## 1 Introduction

Atrial fibrillation (AF) is a prevalent cardiac arrhythmia characterized by the loss of regular, organized electrical activity in the heart, replaced by rapid and irregular fibrillation waves. It represents a significant disruption in normal cardiac electrical activity (1, 2). The estimated global prevalence was 50 million in 2020 (3, 4). AF is associated with a 1.5- to 2-fold increased risk of death (5, 6). In meta-analyses, AF is also associated with increased risk of multiple adverse outcomes, including a 2.4-fold risk of stroke (6), 1.5-fold risk of myocardial infarction (MI) (7), and 1.3-fold risk of peripheral artery disease (6). NVAF refers to atrial fibrillation that occurs aside from cases involving mechanical prosthetic heart valves or those typically associated with moderate to severe mitral stenosis caused by rheumatic heart disease (8). For patients with concomitant valvular disease, the use of warfarin for anticoagulation is recommended. For patients with NVAF, professional guidelines suggest oral anticoagulant therapy for those at increased risk of thromboembolism (9, 10). Past use of warfarin has significantly reduced the risk of stroke in patients with AF (11). Furthermore, warfarin treatment has a narrow therapeutic range, interacts with food and other drugs, and requires regular international normalized ratio monitoring and frequent dose adjustments (12, 13). In recent years, rivaroxaban and dabigatran has been approved for stroke prevention in AF in randomized controlled trials, owing to its noninferiority in both efficacy and safety when compared to warfarin (14, 15).

The most worrisome complication of anticoagulation is bleeding, especially for patients with NVAF with high bleeding risk. In 2010, The European Society of Cardiology (ESC) guidelines for the treatment of AF introduced for the first time the HAS-BLED bleeding risk assessment program. Patients with a HAS-BLED score of ≥3 are considered to be at high bleeding risk (16). Many studies have confirmed that being patients with NVAF with high bleeding risk does not necessarily contraindicate the use of anticoagulant medications (17, 18). Moreover, several studies have comparatively analyzed the clinical benefits of rivaroxaban and warfarin (14, 15, 19–21). In clinical practice, there are many populations receiving oral anticoagulant therapy with rivaroxaban and dabigatran. However, there is limited comparative analysis between rivaroxaban and dabigatran, and these studies have not specifically investigated the East Asian population with high bleeding risk. In clinical settings, patients on Asian with AF with multiple complications tend to have high bleeding risk. Clinicians often lack guidance for medication management in this population. This study specifically conducts a comparative analysis of NVAF patients of East Asian with high bleeding risk, comparing the efficacy and safety risks of rivaroxaban and dabigatran. The findings aim to provide hypothesis generation for further studies.

## 2 Methods

Patients admitted to the Cardiology Department of the First Affiliated Hospital of Zhengzhou University, He Hospital District, and Zhengdong Hospital District from May 31, 2016 to May 31, 2019 were screened through the medical records system. Eleven wards were included (including 2 CCUs) based on the following criteria: (1) Inclusion criteria: (1.1) Diagnosis of AF based on routine electrocardiogram or dynamic electrocardiogram findings; (1.2) Valvular diseases ruled out by echocardiography; (1.3) High risk of bleeding defined as HAS-BLED score ≥3; (1.4) Requiring anticoagulant therapy with rivaroxaban or dabigatran (based on CHADS2-VASc score ≥1); (1.5) Age ≥18 years old. (2) Exclusion criteria: (2.1) Discontinuation of medication without medical advice or failure to adhere to the standard dosage regimen of the study drugs; (2.2) Loss to follow-up during telephone follow-up; (2.3) Switching to other types of oral anticoagulants during the follow-up period.

The study was divided into two groups: the rivaroxaban group and the dabigatran group. (i) Rivaroxaban group, rivaroxaban (Bayer Healthcare Co., LTD., National drug approval J20180077) was used for anticoagulant therapy. The oral dose varied based on the patient's age, weight and creatinine clearance. The standard dosage is 20 mg per day. For patients with low body weight(body mass index less than 18.5), age over 75 years, creatinine clearance between 15 and 49 ml/min or concurrent acute coronary syndrome, adjust the dosage to 15 mg per day. For patients with creatinine clearance between 15 and 49 ml/min and concurrent acute coronary syndrome, adjust the dosage to 10 mg per day, taken once daily at fixed time intervals, with 1 tablet per dose. (ii) Dabigatran group: Anticoagulant therapy was implemented using dabigatran (Boehringer Ingelheim, approval number J20171035, 110 mg × 10 tablets, 150 mg × 10 tablets). The dosage, based bleeding risk, was a fixed oral dose of 110 mg, taken once in the morning and once in the evening, with 1 tablet per dose.

From May 31, 2016 to May 31, 2019, a total of 1,423 NVAF patients who met the inclusion and exclusion criteria were enrolled. Each patient was followed up for 730 days from the start of medication, except in the case of all-cause mortality. After excluding those who were lost to follow-up, did not adhere to medication as per the physician's instructions, or switched to other types of anticoagulants, a total of 1,301 eligible NVAF patients with high bleeding risk remained. Among them, there were 787 cases in the rivaroxaban group, 514 cases in the dabigatran group.

### 2.1 Study endpoints

This retrospective study's primary efficacy endpoint was defined as a composite endpoint including stroke and systemic embolism. The primary safety endpoint was defined as a composite endpoint including fatal or critical organ bleeding. and the secondary efficacy benefit end point was defined as all-cause death and MI. Specifically: Fatal bleeding: Defined as transfusion of whole blood or concentrated red blood cells ≥2 units, or causing a decrease in hemoglobin of ≥2 g/dl. Critical organ bleeding: Defined as bleeding occurring in critical organ sites such as intracranial, spinal, ocular, pericardial, articular, retroperitoneal, or compartment syndrome.

### 2.2 Statistical analysis

The data were analyzed using SPSS 25.0 statistical software. Normally distributed continuous data were expressed as mean ± standard deviation (mean ± SD), and between-group comparisons were made using the *t*-test. Skewed distributed continuous data were represented as [M (P25, P75)], and between-group comparisons were made using the Mann-Whitney *U*-test. Comparison of rates was performed using the chi-square test. The Wilcoxon rank-sum test was used to compare two groups of ordinal data. Hazard ratios (HR), confidence intervals (CIs), and P-values were calculated using a multivariable Cox proportional hazards model. Survival curves were plotted using the Kaplan-Meier method, and survival analysis was conducted using the log-rank test. A *p*-value of ≤0.05 was considered statistically significant.

## 3 Results

The main clinical characteristics of the patients included in the analysis are shown in Table 1. To compare baseline data between the two groups, appropriate statistical methods were employed. Since most clinical characteristics of the two groups were similar.

**Table 1:** Baseline characteristics of the study population (*N* = 1,246).

Characteristic	Rivaroxaban (*n* = 787)	Dabigatran (*n* = 514)	*T*-test or chi-square test	*P* value
Age (years), mean ± SD	71.23 ± 7.73	70.91 ± 8.05	0.723	0.470
Male sex, *n* (%)	461 (58.58)	311 (60.51)	0.480	0.489
^f^CHADS2-VASc score (*n*%)				
1	105 (13.34)	71 (13.81)		
2	170 (21.60)	112 (21.79)		
3	203 (25.79)	129 (25.10)		
4	188 (23.89)	104 (20.23)		
5	73 (9.28)	58 (11.28)	0.331	0.740
6	38 (4.83)	29 (5.64)		
7	10 (1.27)	8 (1.56)		
8	0 (0.00)	2 (0.39)		
9	0 (0.00)	1 (0.19)		
^g^HAS-BLED score (*n*%)				
3	690 (87.67)	442 (86.00)		
4	86 (10.93)	62 (12.06)		
5	10 (1.27)	10 (1.95)	0.903	0.367
6	1 (0.13)	0 (0.00)		
Current baseline characteristics, Mean ± SD				
^d^EF	56.68 ± 9.95	56.20 ± 10.19	0.836	0.403
Systolic blood pressure	162.33 ± 30.63	161.00 ± 25.10	0.812	0.417
Diastolic blood pressure	93.03 ± 18.69	91.89 ± 15.44	1.146	0.252
Hemoglobin	132.59 ± 18.49	132.02 ± 17.06	0.698	0.485
INR	1.53 ± 6.58	1.37 ± 0.80	0.547	0.584
M(P_25_,P_75_)				
Alanine aminotransferase	20.00 (13.00, 32.00)	13.00 (13.00, 28.00)	1.972	0.049
Aspartate aminotransferase	21.00 (17.00, 29.00)	20.00 (17.00, 25.00)	3.493	0.001*
Direct bilirubin	5.50 (3.90, 8.00)	6.20 (4.70, 8.23)	3.802	0.001*
Indirect bilirubin	5.70 (3.70, 8.50)	7.30 (5.10, 10.30)	7.403	0.001*
Alkaline phosphatase	74.00 (61.00, 91.00)	71.00 (59.00, 82.00)	3.503	0.001*
Creatinine	76.00 (65.00, 92.00)	89.30 (75.58, 105.00)	9.002	0.001*
^c^BNP	1,274.00 (639.70, 2,637.24)	1,185.35 (721.91, 2,305.23)	0.723	0.470
Medical history (*n*%)				
Heart failure	245 (31.13)	140 (27.24)	2.262	1.133
Diabetes	153 (19.44)	109 (21.21)	0.602	0.438
Hypertension	583 (74.08)	357 (69.46)	3.315	0.069
Stroke	138 (17.53)	100 (19.46)	0.767	0.381
Thromboembolism^a^	21 (2.7)	13 (2.8)	0.029	0.864
^e^TIA	35 (4.45)	20 (3.89)	0.238	0.626
Vascular disease^b^	37 (4.70)	237 (5.25)	0.202	0.653
History of nonsteroidal drug use	397 (50.44)	2,146 (47.86)	0.831	0.362
Smoking	245 (31.13)	158 (30.74)	0.022	0.881
Alcohol	379 (48.16)	275 (53.50)	3.552	0.059

^a^ Thromboembolism includes pulmonary embolism, organ embolism, and lower limb embolism.

^b^ Vascular disease includes peripheral artery disease, MI, and complex aortic plaques.

^c^ BNP, brain natriuretic peptide.

^d^ EF, ejection fraction.

^e^ TIA, transient ischemic attack.

^f^ CHADS2-VASc, congestive, heart, failure, hypertension, age ≥75 (doubled), diabetes mellitus, stroke (doubled)-vascular disease, age 65–74 and sex category(female).

^g^ HAS-BLED, hypertension, abnormal renal and liver function, stroke, bleeding, labile INRs, elderly, drugs and alcohol.

*Significant value.

**Figure 1:**
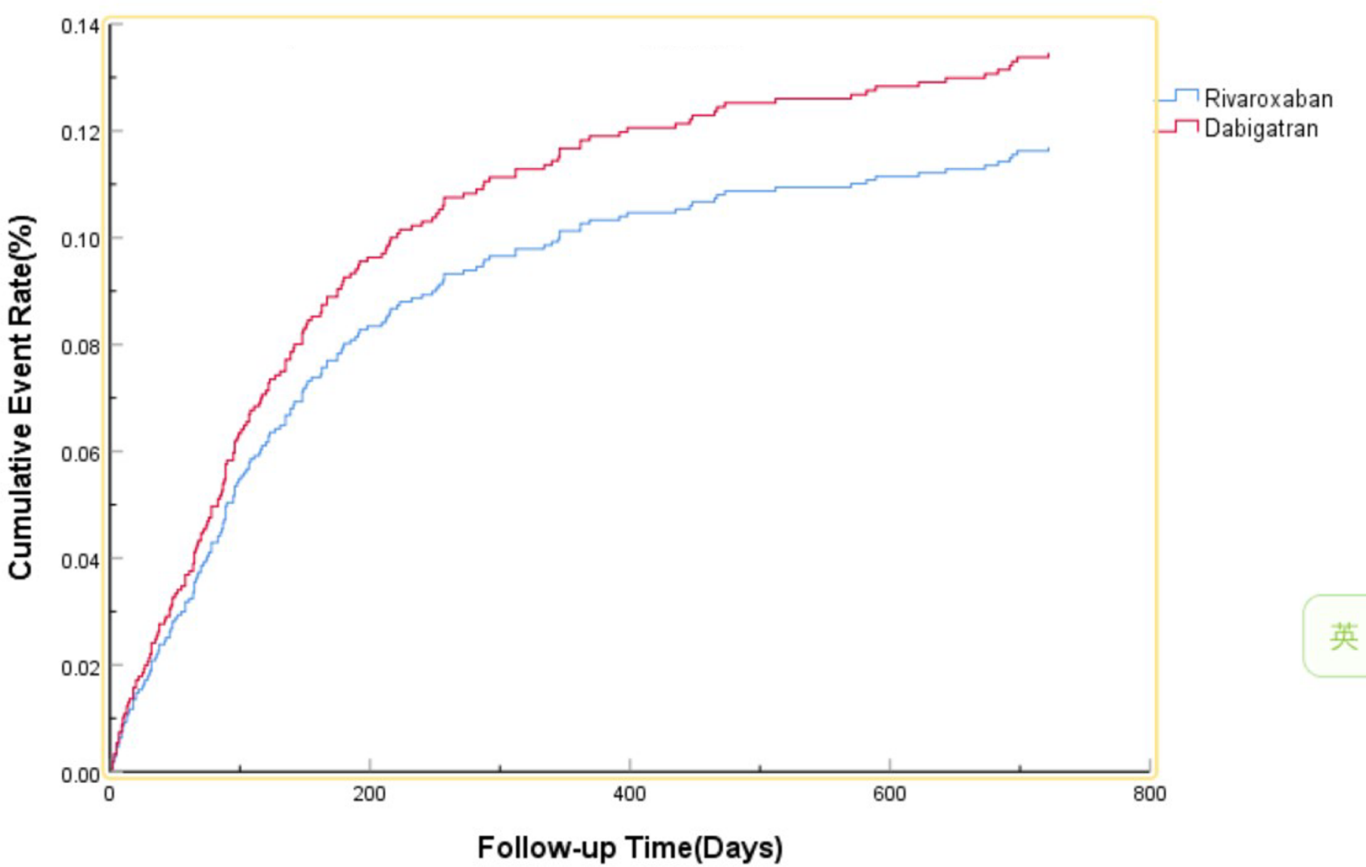
The primary efficacy benefit end point.

**Figure 2:**
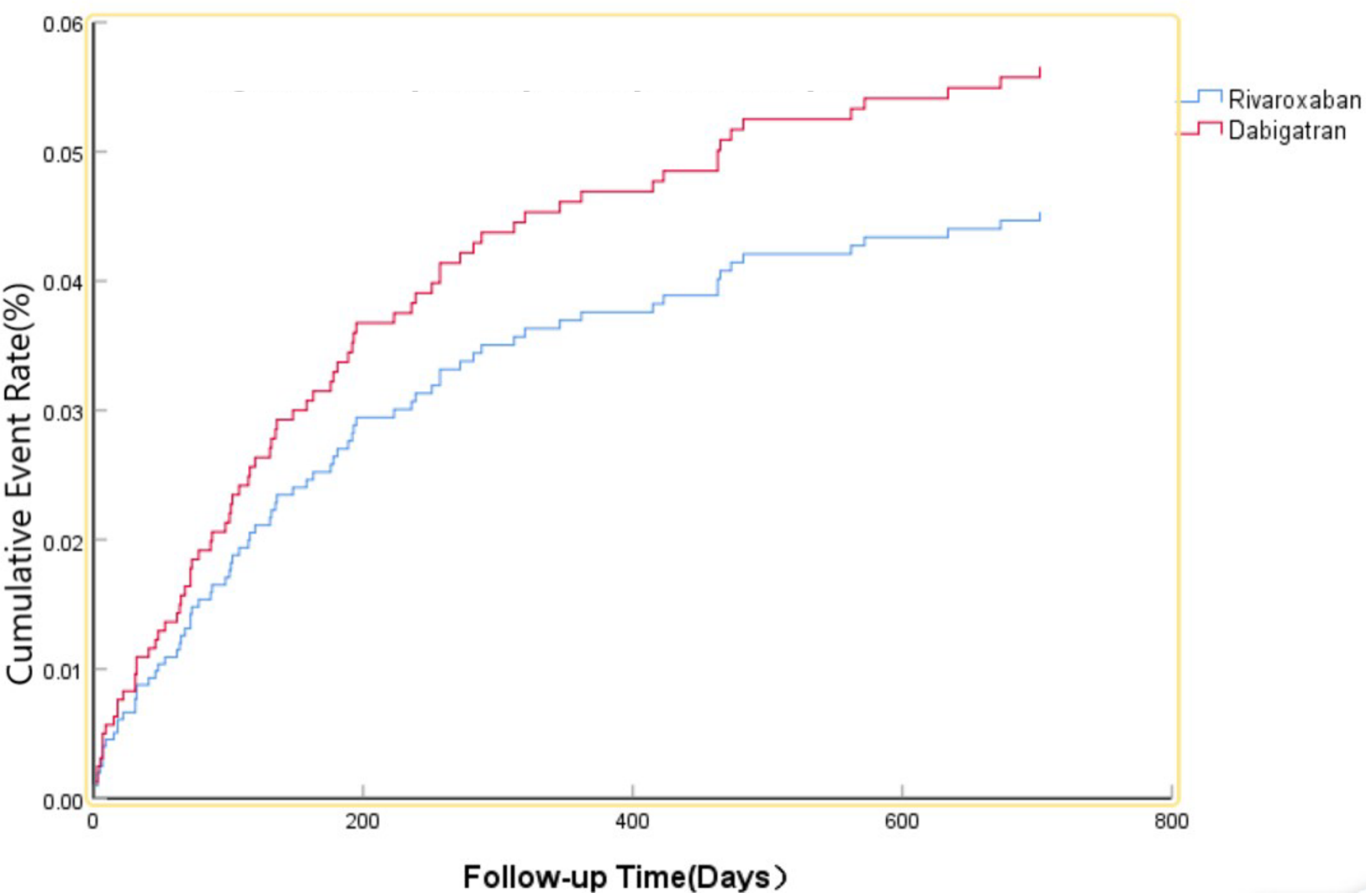
The primary safety risk end point.

The efficacy benefit and safety end points in this study are shown in Table 2. The primary efficacy endpoint occurred in 81 cases (15.76%) in the dabigatran group and 104 cases (13.21%) in the rivaroxaban group, with an HR of 0.860 (95% CI: 0.637–1.162; *P* = 0.327) for the rivaroxaban group. The principal safety end points occurred in 36 cases (7.00%) in the dabigatran group and 49 cases (6.23%) in the rivaroxaban group, with an HR of 0.801 (95% CI: 0.512–1.255; *P* = 0.333) for the rivaroxaban group. This indicates that there was no significant difference between dabigatran and rivaroxaban in preventing stroke and systemic embolism in patients with high bleeding risk NVAF, as shown in Figure 1, nor was there a significant difference in reducing fatal bleeding and critical organ bleeding, as shown in Figure 2.

**Table 2:** Comparison of end point events in the rivaroxaban and dabigatran groups.

Clinical outcome	All (*N* = 1,301, *n*%)	Rivaroxaban (*n* = 787)	Dabigatran (*n* = 514)	Multivariable adjustment OR (95%CI)	*P* value
The primary efficacy endpoint	185 (14.22)	104 (13.21)	81 (15.76)	0.860 (0.637–1.162)	0.327
Stroke^a^	98 (7.53)	57 (7.24)	41 (7.98)	0.909 (0.608–1.135)	0.642
MI^b^	63 (4.84)	32 (4.07)	31 (6.03)	0.668 (0.405–1.102)	0.114
PTE^c^	17 (1.31)	10 (1.27)	7 (1.36)	0.988 (0.375–2.606)	0.981
Lower-extremity thrombosis	29 (2.23)	16 (2.03)	13 (2.52)	1.005 (0.490–2.149)	0.990
All-cause death	64 (4.92)	28 (3.56)	26 (5.06)	0.725 (0.425–1.238)	0.239
Primary safety endpoint	85 (6.53)	49 (6.23)	36 (7.00)	0.801 (0.512–1.255)	0.333
Fatal bleeding^d^	39 (3.00)	21 (2.67)	18 (3.50)	0.729 (0.382–1.393)	0.339
Critical organ bleeding^e^	47 (3.61)	28 (3.56)	19 (3.70)	0.886 (0.492–0.1.594)	0.685
ICH^f^	30 (2.31)	18 (2.29)	12 (2.33)	0.872 (0.412–1.845)	0.720

^a^ Both ischemic and hemorrhagic stroke.

^b^ Myocardial infarction.

^c^ Pulmonary thromboembolism.

^d^ Whole blood transfusion or erythrocyte concentration ≥2 units or hemoglobin reduction ≥2 g/dl.

^e^ Bleeding from any of the following anatomical sites: intracranial, spinal, eye, pericardium, joint, retroperitoneal, or muscular compartment syndrome.

^f^ Intracranial hemorrhage.

Secondary efficacy and benefit endpoints: there were 26 cases (5.06%) of all-cause death in the dabigatran group and 28 cases (3.56%) in the rivaroxaban group, with an HR of 0.725 (95% CI: 0.425–1.238; *P* = 0.239) for the rivaroxaban group. There were 31 cases (6.03%) of MI in the dabigatran group and 32 cases (4.07%) in the rivaroxaban group, with an HR of 0.668 (95% CI: 0.405–1.102; *P* = 0.114) for the rivaroxaban group. This indicates that there was no significant difference between dabigatran and rivaroxaban in preventing all-cause death and MI.

## 4 Discussion

We compared fixed-dose dabigatran and rivaroxaban for patients with NVAF and a risk of stroke. In terms of primary efficacy endpoints, there was no significant difference between dabigatran and rivaroxaban in treating stroke and systemic embolism. In the RELY study (14), the 150 mg dose of dabigatran was superior to warfarin in treating stroke or non-central embolism, and the 110 mg dose was superior to warfarin in terms of major bleeding. In the ROCKET-AF study (15), rivaroxaban was non-inferior to warfarin in preventing stroke and systemic embolism. In the ARISTOPHANES study (20), both dabigatran and rivaroxaban were associated with lower rates of stroke and systemic embolism compared to warfarin, but there was no significant difference between dabigatran and rivaroxaban. In our study, dabigatran and rivaroxaban also showed no significant difference, indicating similar therapeutic effects in populations with high bleeding risk. This is also broadly similar to the results of studies involving catheter ablation in patients with AF (22–25). The most concerning complication of anticoagulant therapy is the risk of bleeding, especially fatal and critical organ bleeding. In previous studies (14, 15, 20), it has been demonstrated that Rates of intracranial bleeding and critical organ bleeding were higher with warfarin than with either dabigatran or rivaroxaban. Dabigatran has a lower incidence of major bleeding compared to rivaroxaban (20). However, in this study, there was no significant difference observed between dabigatran and rivaroxaban in reducing fatal and critical organ bleeding. One possible explanation is that the majority of the population included in this study had a high risk of bleeding, prompting clinicians to administer medication with greater caution, often opting for lower doses compared to previous studies. Additionally, the unique genetic makeup of the Asian population may also play a role (26–28), which warrants further evidence-based medical research for validation. The final possible explanation is that the little number of events in the population may necessitate further studies with a large sample size.

The primary secondary efficacy endpoint of all-cause death and MI also showed no significant difference between the two groups. However, a phenomenon was observed during the Phase III clinical trials of dabigatran, where a slight increase in the incidence of MI was noted among patients taking the medication (14). Guidelines also recommend against prioritizing dabigatran for patients at higher risk of coronary artery disease or MI (29). This aspect requires further research for confirmation.

The study population comprised hospitalized patients receiving treatment at the hospital, and all data were derived from the hospital's case system and patient follow-up processes. Compared to previous real-world studies, both rivaroxaban and dabigatran are equally effective, with dabigatran showing an advantage in reducing major bleeding (30–32). However, in this study, there was no significant difference between the two groups in terms of reducing major bleeding. This could be related to the fact that all participants in this study had high bleeding risk, differences in drug dosing, and possibly the number of participants included. This closely mirrors real-world clinical treatment scenarios, providing valuable insights for actual clinical medication practices.

This retrospective observational study has several limitations. Firstly, due to the inherent limitations of retrospective studies, only statistical associations can be inferred, not causal relationships. Secondly, our data were obtained from hospitalized patients in the Department of Cardiology at the First Affiliated Hospital of Zhengzhou University, so the conclusions may be most representative for the Chinese population in East Asia. Finally, given the large number of statistical tests, especially interaction tests, we cannot rule out the possibility of type I errors.

## 5 Conclusion

Overall, In NVAF patients with high bleeding risk, there was no significant difference between dabigatran and rivaroxaban in preventing stroke and systemic embolism. There was also no significant difference between dabigatran and rivaroxaban in reducing fatal and critical organ bleeding.

## Data Availability

The original contributions presented in the study are included in the article/Supplementary Material, further inquiries can be directed to the corresponding author.
